# Organoid in dentistry: Models for oral biology and disease

**DOI:** 10.1016/j.jds.2025.05.002

**Published:** 2025-05-15

**Authors:** Tomomi Sano, Ting-Yi Renn, Takashi Kanematsu, Ming-Shou Hsieh, Chia-Chen Hsu, Wei-Jen Chang

**Affiliations:** a Department of Cell Biology, Aging Science, and Pharmacology, Division of Oral Biological Sciences, Faculty of Dental Science, Kyushu University, Fukuoka, Japan; b School of Dentistry, College of Oral Medicine, Taipei Medical University, Taipei City, Taiwan; c School of Oral Hygiene, College of Oral Medicine, Taipei Medical University, Taipei City, Taiwan; d Department of Dentistry, Taipei Medical University-Shuang Ho Hospital, New Taipei City, Taiwan

**Keywords:** Organoid, Stem cell, Tooth, Salivary glands, Periodontal ligament, Bone

## Abstract

Cell lines and animal models have long been used as essential tools in studies targeting the oral cavity, offering valuable insights into various oral diseases. Each of these research models provides its advantages, such as ease of manipulation in cell lines and the ability to replicate whole–organ interactions in animal models. However, conventional models often have limited native phenotypic features, which do not fully capture the complexity of the human oral cavity. In response to these limitations, organoid technologies have recently been developed and emerged as a promising alternative. Organoids, which are widely applied to mimic the complexity of oral tissues, such as tongue (including taste buds), tooth germs, teeth, salivary glands, periodontal ligament, bone, and oral squamous cell carcinoma, offer a more proper model for studying oral biology and disease. Key signaling pathways, including Wnt/β-catenin, transforming growth factor beta (TGF-β) (bone morphogenetic protein (BMP), and fibroblast growth factor (FGF), have been demonstrated to play important roles in expansion and differentiation of oral organoids. These advancements have opened new avenues for understanding the development and pathology of oral cavity. Therefore, we summarize current novel oral organoid culture strategies and their application, providing a deeper understanding of the biology of the oral cavity and the pathophysiology of oral diseases.

## Introduction

The oral cavity is composed of teeth, periodontal tissue, tongue, and salivary glands, and is responsible for functions such as speech, chewing, and swallowing. Cell lines that have been universally used for research in the oral cavity are the most common disease models, with the advantages of time efficiency, cost-effectiveness, and ease of manipulation. However, they cannot reproduce the original phenotypic features, complex cell types, or 3D structures of the oral cavity. Animal disease models, genetically engineered mouse models, and patient-derived xenograft (PDX) models have the potential to mimic the original histological and genetic features of oral tissues and oral diseases.^1^, ^2^, ^3^, ^4^ However, these models are time-consuming and expensive, limiting their application for high-throughput screening and precise personalized therapies. To overcome the deficiencies of these models, there is an urgent need to establish ideal *in vitro* models that can expand rapidly and realistically mimic the characteristics of oral tissues.

Organoids are excellent *in vitro* models that can maintain the genetic and phenotypic characteristics of native human tissues. Organoids derived from normal or tumor tissues have been validated to reproduce realistic microstructures and biological functions. Compared to cultured cells and spheroids, organoids exhibit anatomically and functionally similar features to *in vivo* organs, and it is possible to construct organoids from both normal or diseased tissues.^5^ Several oral organoids have been successfully constructed, including taste bud organoids, tooth embryo organoids, and salivary gland organoids.

**Figure 1:**
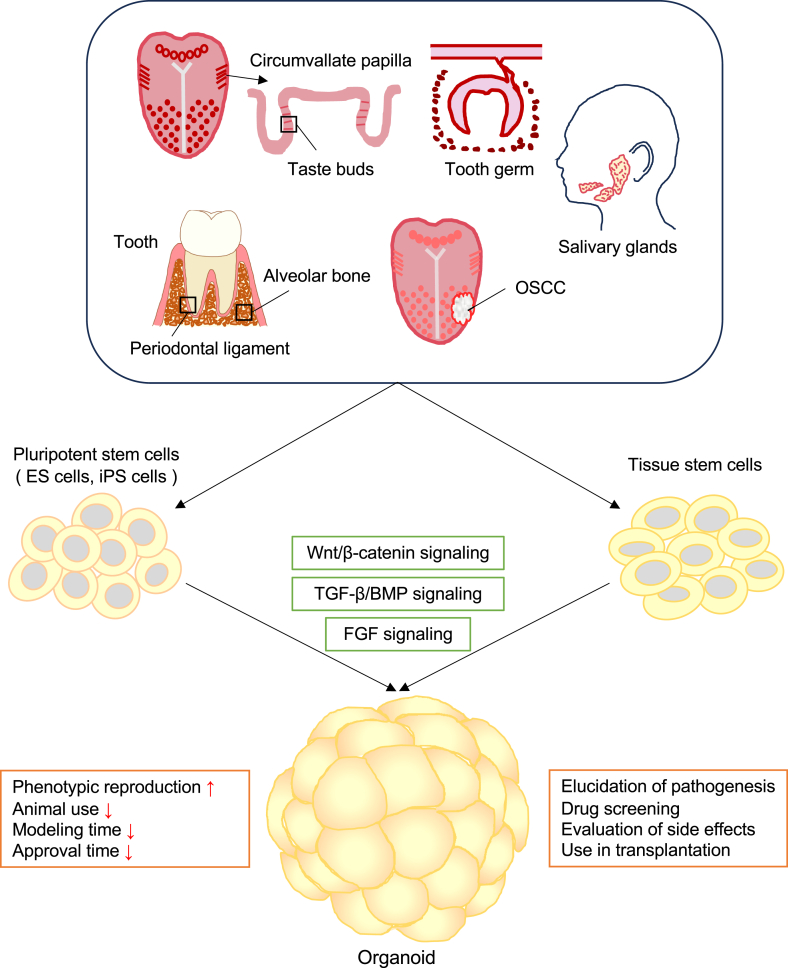
Oral organoids are created by extracting or establishing multiple tissue stem cells or pluripotent stem cells from the oral cavity and culturing them under appropriate conditions. The resulting organoids are expected to have a variety of applications. OSCC: oral squamous cell carcinoma, ES cells: embryonic stem cells, iPS cells: induced pluripotent stem cells, BMP: bone morphogenetic protein, TGF-β: transforming growth factor beta, FGF: fibroblast growth factor.

This review describes recent advances in oral organoid models that recapitulate natural oral tissues *in vitro*, showing great value and potential in modeling human oral diseases. These models are useful for studying the molecular mechanisms related to oral cavity diseases and for drug screening. We will summarize various new oral organoid culture systems and their unique applications, as well as describe the key signaling pathways that support organoid proliferation and differentiation. Lastly, we will focus on the challenges and future developments of oral organoids to realize their potential in the study of human oral pathophysiology (Fig. 1).

## Development of oral organoids

Organoids are constructed from tissue stem cells and pluripotent stem cells (induced pluripotent stem (iPS) cells, embryonic stem (ES) cells).^6^ The potential for differentiation from stem cells into diverse tissues and organs is anticipated through stimulation with growth factors and other factors (as shown in Table 1).

**Table 1:** A summary table of culture conditions for the oral organoids.

Organoid	Origin cell	Key factor in organoid medium	Ref.
Tooth germ	Human ESC and iPSC	N2, RA, BMP4	12
Tooth	Human dental pulp stem cell	l-ascorbic acid, β-glycerophosphate, Dexamethasone	13
Tooth	Human dental follicle stem cell and dental pulp stem cell	A83–01, B27, Cholera toxin, FGF2, FGF8, FGF10, l-glutamine, IGF1, N2, N-acetylcysteine, Nicotinamide, Noggin, RSPO, SB202190, SHH, WNT3A	14
Tooth, PDL	Human dental pulp stem cell and periodontal ligament stem cell	FGF2, Ascorbic acid, Dexamethasone, β-glycerophosphate, TGF-β	16
Salivary glands	Human salivary gland stem cell	Fungizone, EGF	17
Salivary glands	Human dental follicle stem cell	N2, EGF, FGF2	18
Salivary glands	Human iPSC	N2, B27, FGF7, FGF10	20
PDL	Human dental follicle stem cell	B27, N-acetylcysteine, Nicotinamide, EGF, A83-01, FGF10, FGF2, PGE2, CHIR-99021, RSPO, Noggin	21
OSCC	Human OSCC sample	B27, N-acetylcysteine, Nicotinamide, EGF, FGF10, FGF2, A83-01, PGE2, CHIR-99021, RSPO, Forskolin, Noggin, Y-27632	25
OSCC	Human OSCC sample	B27, N-acetylcysteine, Nicotinamide, EGF, FGF10, FGF2, PGE2, CHIR-99021, Forskolin, Noggin, RSPO	26

ESC: embryonic stem cell, iPSC: induced pluripotent stem cell, RA: retinoic acid, BMP4: bone morphogenetic protein 4, A83-01: inhibitor of transforming growth factor beta receptor 1 (TGF-β1), FGF: fibroblast growth factor, IGF1: nsulin-like growth factor 1, RSPO: R-spondin, SB202190: p38 MAPK inhibitor, SHH: Sonic hedgehog, PDL: periodontal ligament, EGF: Epidermal growth factor, PGE2: prostaglandin E2, CHIR-99021: inhibitor of glycogen synthase kinase 3 (GSK-2), OSCC: oral squamous cell carcinoma, Y-27632: inhibitor of rho-associated protein kinase (ROCK).

### Taste bud organoids

Taste buds are bud-like organs located mainly on the tongue and soft palate that are responsible for the perception of taste. Each taste bud is composed of approximately 50–150 cells with various properties, some of which express taste receptors and function as taste receptor cells (taste cells) that respond to taste stimuli.^7^ The cells within the taste buds are derived from epithelial cells, but the taste cells that respond to taste stimuli express voltage-gated Na^+^ channels and generate action potentials, thus exhibiting neuron-like properties. Leucine-rich repeat-containing G protein-coupled receptor 5 (Lgr5) is a G protein-coupled receptor that plays a pivotal role in embryonic development and serves as a marker for adult stem cells in various tissues and organs.^8^ Monocultured Lgr5^+^ cells give rise to gustatory cells, and a taste bud organoid, consisting of a multilayered epithelium with stem/progenitor cells in the outer layer and gustatory cells in the inner layer, was established using vallate papilla tissue.^9^ It has also been shown that Lgr6, a homologous gene of Lgr5, labels adult gustatory stem/progenitor cells in the anterior tongue. Like Lgr5^+^ cells, isolated Lgr6^+^ cells can also generate mature gustatory cells and construct organoids.^10^ Subsequently, Adpaikar et al. fine-tuned conventional taste bud organoids, which had inaccessible sites of taste receptor cells, had been observed by modifying apical-basal polarity and established a suspension culture method to form accessible sites for taste receptor cells.^11^ Compared to conventional Matrigel-embedded organoids, these organoids showed differentiation and renewal rates comparable to those of taste buds *in vivo*. Because of their accessibility to taste receptor cells, calcium imaging can be directly applied to assess taste responses. Furthermore, the organoids are genetically recombinable. Suspension-cultured taste bud organoids are harmonically integrated with the receptor tongue epithelium and maintain taste receptor cells and gustatory innervation capabilities, potentially providing an efficient model for taste studies, including taste bud development, regeneration, and transplantation.

### Tooth embryo and tooth organoids

Teeth are produced by tooth embryos. Cai et al. differentiated human urine-induced pluripotent stem cells into epithelial sheets, which were then recombined with dental mesenchyme from E14.5 mice to create dental embryo organoids.^12^ The epithelial sheets differentiated into enamel-secreting ameloblasts and exhibited physical properties, such as elastic modulus and hardness, similar to those found in normal human teeth. Jeong et al. also established a method to produce dentin-pulp-like organoids with both stem cell and dentinoblast characteristics from human dental pulp stem cells, which are mesenchymal cells.^13^ In this organoid, dentinoblasts were primarily found in the outer portion, while stem cells were located in the inner portion. Subsequently, the first human tooth organoid was developed that was based on human teeth, and exhibited an epithelial stem cell phenotype and differentiation potential.^14^ Dental follicle tissue isolated from unerupted wisdom teeth efficiently generated epithelial organoids capable of long-term proliferation. These organoids exhibited a tooth epithelial stem cell phenotype similar to the epithelial cell remnant of Malassezia (ERM), a compartment containing epithelial stem cells. Exposure of the organoids to epidermal growth factor induced transient proliferation and eventual epithelial-mesenchymal transition, closely mimicking events occurring in the ERM *in vivo*. It was then reported that a tooth organoid model was created from both molars and incisors of mice.^15^ The resulting organoids are expandable over time, reproduce tooth-specific features, and exhibit differentiation characteristics of dental epithelial stem cells (DESCs) both *in vivo* and *in vitro*. This *in vitro* differentiation is further enhanced by the presence of dental mesenchymal (pulp) stem cells, reflecting the important epithelial-mesenchymal crosstalk that occurs during tooth development, providing a new and powerful tool to explore and contrast the biology and development of molars and incisors. Calabrese et al. showed that dental pulp stem/progenitor cells (DPSCs) and periodontal ligament stem/progenitor cells (PDLSCs) could take advantage of their innate self-organizing capacity to design organoids that resemble complete tooth roots. This root organoid was shown to be composed of two biochemically distinct calcified tissues characterized by dentin-like and cementum-like structures, respectively.^16^

### Salivary gland organoids

Salivary glands are tissues that secrete saliva into the oral cavity. Saliva has digestive, antibacterial, and protective effects on the oral mucosa and plays an important role in maintaining the oral environment. It has been reported that salivary gland organoids can be generated from salivary gland-derived stem/progenitor cells^17^ and dental vesicle stem cells.^18^ However, these organoids showed different marker expression patterns compared to those observed in real tissues. Following this, a method for generating functional salivary gland organoids from mouse embryonic stem cells was reported.^19^ When th``ong-term cultures of adult salivary gland organoids, consisting of salivary gland acinar cells, conduit cells, and myoepithelial cells, from all types of major salivary glands in both mice and humans.^20^ Three-dimensional salivary gland organs were regenerated from oral mucosal epithelium induced from human iPS cells, achieved through stepwise reproduction of the differentiation process of salivary glands. The salivary gland organoids derived from these iPS cells resembled human embryonic salivary gland primordia in terms of both morphological characteristics and gene expression analysis.

### Other organoids

The periodontal ligament (PDL) is a thin membrane that covers the tooth root and connects the tooth to the alveolar bone. As periodontitis progresses, the periodontal ligament is destroyed, and the alveolar bone is resorbed by toxins and inflammatory cytokines released by bacteria associated with periodontal disease. Therefore, the application of periodontal ligament organoids and alveolar bone organoids in the field of periodontal tissue regeneration is highly anticipated. For periodontal ligament organoids, expandable organoid cultures from human periodontal ligament which show typical characteristics of epithelial stem cells have been established.^21^ As mentioned earlier, it has also been revealed that periodontal ligament stem/progenitor cells (PDLSCs) can self-organize into PDL organoids.^16^ Numerous new protocols have also been developed for bone organoids.^22^, ^23^, ^24^ Using these methods, research into bone regeneration therapy is accelerating, in which bone organoids constructed by combining iPS cells or mesenchymal stem cells with scaffold materials through tissue engineering are transplanted into patients to treat alveolar bone deficiency.

Oral squamous cell carcinoma (OSCC) is the most common form of head and neck cancer. Patient-derived organoids have become a promising cell culture system for disease modeling and precision medicine. Zhang et al. used 3D technology to screen and optimize the culture medium to construct a human-derived OSCC organoid model. The organoids were subjected to morphological validation, immunofluorescence analysis, tissue origin validation, and short tandem repeat (STR) sequencing to confirm the consistency between the organoids and the source tissue.^25^ In addition, tumor angiogenesis is influenced by various cell types in the tumor microenvironment (TME), including cancer cells and cancer-associated fibroblasts (CAFs). Therefore, Holkom et al. constructed OSCC organoids to investigate the mechanisms underlying the regulation of angiogenesis by the TME in OSCC.^26^

## Key signals regulating proliferation and differentiation of oral organoids

Understanding the signaling pathways that regulate stem cell fate is essential for establishing organoid cultures.

### Wnt/β-catenin signaling

In taste buds, β-catenin signaling has been reported to be involved in determining taste cell fate.^27^ Both Hedgehog and Wnt/β-catenin are required for taste homeostasis in adult mice.^28^^,^^29^ SRY-Box transcription factor 2 (SOX2) is an important regulator of homeostasis in many adult epithelia,^30^ and Wnt/β-catenin signaling promotes taste cell differentiation in organoids derived from progenitor cells with high levels of SOX2 expression.^31^ Maimets et al. also found that in cultures of salivary gland organoids, stem cells in the salivary gland lumen expressed nuclear β-catenin, reported that Wnt signaling is active.^32^ They showed that inhibition of Wnt signaling abolished long-term organoid cultures, concluding that Wnt signaling is a critical driving force of salivary gland stem cells. Additionally, it has been shown that multiple signaling pathways, including Wnt, bone morphogenetic protein (BMP), sonic Hedgehog (SHH), and Notch, are involved in the generation of gustatory cells.^33^ Of these four signaling pathways, Wnt and SHH signaling are activated during taste bud organoid growth, while BMP and Notch signaling are inactivated. Inhibition of the Notch signaling pathway by LY411575 promotes the generation of taste receptor cells in the cardinal papillary organoids. This inhibition of Notch signaling, leads to the formation of taste receptor cells in the cardinal papillary organoids, which are then activated by Wnt and SHH signaling, regulates multiple signaling pathways, thus the generation of receptor cells in taste bud organoids is regulated by multiple signaling pathways.^34^

### TGF-β/BMP signaling

Transforming growth factor – beta (TGF-β) and BMP signaling play an important role in regulating the ability of dental epithelial cells to proliferate and respond to differentiation during tooth development.^35^ As an example of the application of TGF-β in organoids made from human gingival-derived stem cells, TGF-β promoted differentiation while maintaining cell viability. This was confirmed by alkaline phosphatase activity and mRNA expression.^36^ In the tooth organoids developed by Hemeryck et al., epithelial stem cell organoids can develop a differentiation process to enamel blast cells, which can be further promoted by TGF-β and inhibited by TGF-β receptor antagonism.^14^ Wang et al. also verified that signal peptide-CUB-EGF domain-containing protein 3 (SCUBE3), which plays a vital role in tooth development, regulates mesenchymal dentoblast differentiation.^37^ SCUBE3 was shown to promote human DPSCs proliferation and migration through the TGF-β/Smad pathway. Furthermore, in organoid models, SCUBE3 has been suggested to induce dentoblast differentiation of human DPSCs via the BMP2/Smad pathway. In addition, activation of activin receptor kinase (Alk) signaling, which includes both BMP and TGF-β pathways, has been associated with salivary gland dysfunction.^38^^,^^39^ Therefore, inhibition of Alk signaling is required for the formation of the successful formation of human salivary gland-derived organoids.^40^

### Other signals

Endogenous and exogenous signals regulate the stemness and proliferation of epithelial tissues.^41^ Fibroblast growth factor (FGF) signaling serves as an extrinsic signal in several epithelial stem cell niches.^42^ Seubert et al. used organoid technology to identify three FGF ligands (FGF1, FGF7, and FGF10) as site-specific niche factors for the dorsal and ventral tongue.^43^ It has been shown that calcified dental epithelial organoids can be generated from dental epithelial stem cells derived from mouse incisor tissue by treatment with a combination of Wnt, BMP, FGF, and Notch signaling regulators.^44^ Retinoic acid signaling has also been shown to induced lumen formation in a study demonstrating that a salivary gland organoid culture system can reproduce lumen formation in mouse submandibular glands.^45^

## Biomedical applications of oral organoids

In recent years, oral organoid culture techniques derived from adult stem/progenitor cells and pluripotent stem cells (iPS cells, ES cells) have been greatly developed and improved.^46^ These techniques preserve to some extent the main functions and characteristic structures of the corresponding organs. Furthermore, by using cells isolated from patients, oral organoids can reproduce certain diseases, such as maxillofacial tumors and tooth dysplasia. This makes them as a versatile model for studying oral and maxillofacial tissue development, pathology, and regeneration *in vitro*. The following are some of the most important features of these models.

A protocol for generating periodontal ligament organoids from epithelial stem cells derived from human dental follicles has been developed for viral infection and drug screening.^21^ A novel periodontal ligament organoid model for studying severe acute respiratory syndrome coronavirus (SARS-CoV-2) was introduced, and the antiviral capacity of ovatodioride (OVA) was investigated. The use of this periodontal ligament organoid model highlighted the potential of OVA as a small molecule therapeutic agent that inhibits the ability of the virus to bind to the neuropilin-1 receptor on host cells, and proved it to be an ideal *in vitro* model for studying SARS-CoV-2. Studies using salivary gland organoids derived from human iPS stem cells also confirmed the infection and replication of SARS-CoV-2 in the salivary glands. These salivary gland organoids may provide a promising model for investigating the role of the salivary gland as a reservoir of the virus.^20^ In addition, an oral epithelial organoid air-liquid interface (ALI) culture model has been developed to recapitulate Kaposi's sarcoma-associated herpesvirus (KSHV) infection in the oral cavity. Unlike 2D cell cultures, 3D oral epithelial organoid ALI cultures allow lytic replicating cells to concentrate on the surface layer of the epithelial organoid, producing high levels of spontaneous KSHV soluble replication. In this model, KSHV undergoes epithelial differentiation-dependent spontaneous lytic replication, creating a unique cell population with different viral gene expression.^47^

Oral organoids are also used in drug screening to assess drug efficacy and toxicity. Dexamethasone, known for its anti-inflammatory and immunomodulatory properties, is widely used in clinical practice. However, studies on the characterization mechanisms and side effects of dexamethasone in salivary glands are lacking. To address this, the therapeutic effect of dexamethasone in the treatment of obstructive sialadenitis and its associated side effects were investigated using salivary gland organoids. Dexamethasone inhibited the induction of proinflammatory cytokines in an inflammatory model of salivary gland organoids induced by inflammatory agents. Macrophages cultured in inflammatory agent-treated medium from salivary gland organoid cultures showed inflammation-induced polarization. However, treatment dexamethasone shifted macrophages toward an anti-inflammatory phenotype. Despite these positive effects, high-dose or long-term dexamethasone treatment induced adenoductalization and resulted in adverse effects.^48^ Botulinum neurotoxin (BoNT) is a substance used in the treatment of chronic fluorosis, muscular dystonia, and has cosmetic applications. Due to the potency of BoNT at low doses, it is important to measure its effectiveness accurately. An Neuro2a (N2a) neuron-salivary gland organoid (SGO) co-culture system was used to measure BoNT potency, serves as a platform for BoNT efficacy testing. BoNT treatment altered the expression of salivary gland secretory cell-related in the organoids.^49^ Additionally, cisplatin, a chemotherapy drug commonly used to treat various cancers, has been shown to significantly impact taste perception. Ren et al. used taste bud organoids to determine the effects of cisplatin on taste cell homeostasis and taste function. Cisplatin inhibited proliferation of the cardinal papilla, promoted apoptosis, and severely impaired taste function and receptor cell generation. These findings suggest cisplatin as potential therapeutic targets and strategies for treating taste dysfunction.^50^

### Summary and perspectives

**Figure 2:**
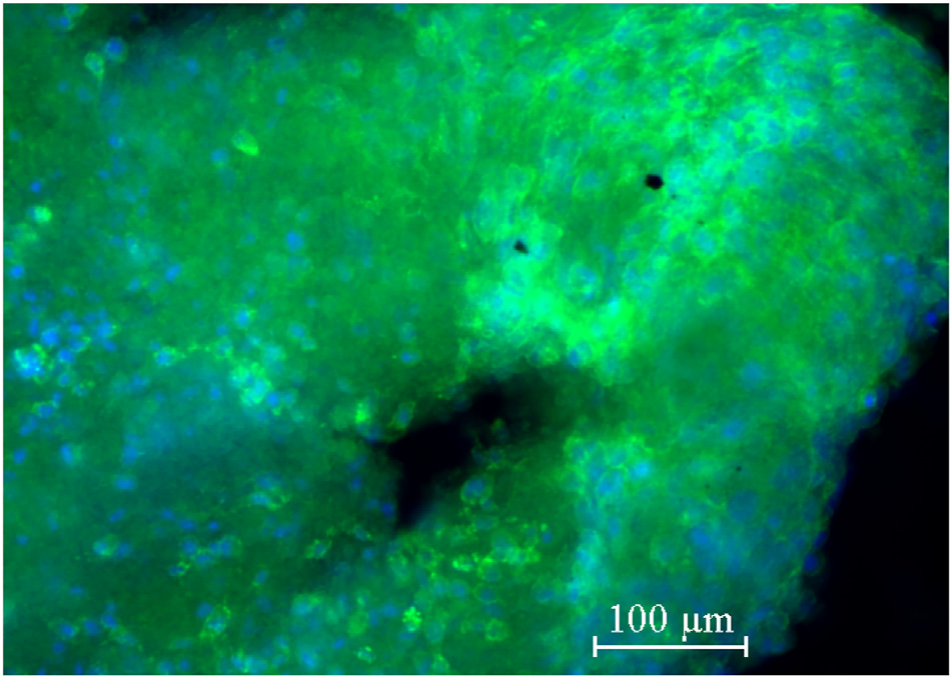
Immunofluorescence staining images of osteo-like organoid derived from MG-63 osteoblast-like cells, which were stained phalloidin (green) to visualize actin filaments, and counterstained with DAPI (blue) to label the cell nuclei.

Oral organoids provide an excellent *in vitro* model for simulating complex features of oral tissue and the effects of oral diseases. A major difference between cells in 2D and 3D cultures is their morphology. In 2D cultures, cells stabilize into a shape based on the orientation of integrin-mediated cell adhesion to the extracellular matrix (ECM), so adhesion occurs on one side of the cell. In contrast, in 3D cultures, adhesion occurs on the entire surface of the cell, and the cell is surrounded by a complex 3D-structured ECM (Fig. 2). The use of 3D culture systems has accelerated mechanistic studies of salivary gland stem cell self-renewal^51^ and regeneration after injury.^52^ In addition, oral organoids can be used to discover targets and side effects of drugs applied in the oral cavity, leading to safer drug development. Many researchers have recently attempted to realize organoid transplantation therapy^53^^,^^54^ and oral organoids could be a source of transplant tissue to replace damaged areas in regenerative medicine. However, such studies have certain limitations. The possibility of severe allograft rejection in xenograft experiments limits the extent of transplantation. Furthermore, even in allogeneic transplantation, stem cell function, especially regenerative capacity, has been reported to decline with age,^55^ and the quality of stem cells, due to the age of the donor from which they are derived may be problematic. In the future, as further research resolves these issues and technological advances are made in establishing multi-tissue organoids and co-culture systems, human oral organoids may begin to more accurately mimic the biological conditions of the human oral cavity. They have the potential to validate findings from previous cell lines and animal models and accelerate our understanding of mechanisms in oral development, homeostasis, and disease processes. We believe that human oral organoids hold great promise for future oral bioscience research and provide a valuable and powerful tool for understanding the complex biological processes that occur in the oral cavity.

## Declaration of competing interest

The authors have no conflicts of interest relevant to this article.
